# Optimized De Novo Eriodictyol Biosynthesis in *Streptomyces albidoflavus* Using an Expansion of the Golden Standard Toolkit for Its Use in Actinomycetes

**DOI:** 10.3390/ijms24108879

**Published:** 2023-05-17

**Authors:** Patricia Magadán-Corpas, Suhui Ye, Álvaro Pérez-Valero, Patrick L. McAlpine, Paula Valdés-Chiara, Jesús Torres-Bacete, Juan Nogales, Claudio J. Villar, Felipe Lombó

**Affiliations:** 1 Research Group BIONUC (Biotechnology of Nutraceuticals and Bioactive Compounds), Departamento de Biología Funcional, Área de Microbiología, Universidad de Oviedo, 33006 Oviedo, Spain; magadanpatricia@uniovi.es (P.M.-C.); yesuhui@uniovi.es (S.Y.); apv.moratalla@gmail.com (Á.P.-V.); mcalpineatsantaclara@gmail.com (P.L.M.); uo269925@uniovi.es (P.V.-C.); cjvg@uniovi.es (C.J.V.); 2 IUOPA (Instituto Universitario de Oncología del Principado de Asturias), 33006 Oviedo, Spain; 3 ISPA (Instituto de Investigación Sanitaria del Principado de Asturias), 33006 Oviedo, Spain; 4 Department of Systems Biology, Centro Nacional de Biotecnologia, CSIC, 28049 Madrid, Spain; jtbace8@gmail.com (J.T.-B.); jnogales@cnb.csic.es (J.N.); 5 Interdisciplinary Platform for Sustainable Plastics towards a Circular Economy-Spanish National Research Council (SusPlast-CSIC), 28040 Madrid, Spain

**Keywords:** eriodictyol, naringenin, flavonoid, *Streptomyces*, synthetic biology, Golden Standard, CRISPR-Cas9, SEVA, chimaera

## Abstract

Eriodictyol is a hydroxylated flavonoid displaying multiple pharmaceutical activities, such as antitumoral, antiviral or neuroprotective. However, its industrial production is limited to extraction from plants due to its inherent limitations. Here, we present the generation of a *Streptomyces albidoflavus* bacterial factory edited at the genome level for an optimized de novo heterologous production of eriodictyol. For this purpose, an expansion of the Golden Standard toolkit (a Type IIS assembly method based on the Standard European Vector Architecture (SEVA)) has been created, encompassing a collection of synthetic biology modular vectors (adapted for their use in actinomycetes). These vectors have been designed for the assembly of transcriptional units and gene circuits in a plug-and-play manner, as well as for genome editing using CRISPR-Cas9-mediated genetic engineering. These vectors have been used for the optimization of the eriodictyol heterologous production levels in *S. albidoflavus* by enhancing the flavonoid-3′-hydroxylase (F3’H) activity (by means of a chimera design) and by replacing three native biosynthetic gene clusters in the bacterial chromosome with the plant genes *matBC* (involved in extracellular malonate uptake and its intracellular activation into malonyl-CoA), therefore allowing more malonyl-CoA to be devoted to the heterologous production of plant flavonoids in this bacterial factory. These experiments have allowed an increase in production of 1.8 times in the edited strain (where the three native biosynthetic gene clusters have been deleted) in comparison with the wild-type strain and a 13 times increase in eriodictyol overproduction in comparison with the non-chimaera version of the F3′H enzyme.

## 1. Introduction

Flavonoids are biologically active phytochemicals of enormous nutraceutical, pharmaceutical and agroindustrial relevance due to the vast array of properties they display [1,2,3]. Specifically, eriodictyol ((2S)-2-(3,4-dihydroxyphenyl)5,7-dihydroxy-2,3-dihydrochromen-4-one), a natural flavanone, has been reported to possess antioxidant [4], antitumoral [5,6,7], antiviral [8], neuroprotective [9,10], anti-inflammatory [11], cardioprotective [12], anti-diabetes [13], anti-obesity [14] and immunomodulatory [15] properties. Apart from its evident therapeutic application, eriodictyol stands out for its application in other fields, such as cosmetics (as a photoprotector) [16] and the food industry (as a bitter-taste masking agent) [17,18].

Eriodictyol is naturally found in citrus fruits, some vegetables and medicinal plants such as *Eriodictyon californicum* [19,20]. However, its extraction from plants presents some major drawbacks, such as its low concentration in plant tissues and the expensive purification and separation procedures from the complex plant extracts [21]. 

As a promising industrial alternative, the development of metabolic engineering and synthetic biology tools has facilitated the heterologous production of eriodictyol in microbial cell factories, such as *Escherichia coli* [21,22], yeast [23,24], *Corynebacterium glutamicum* [25] and *Streptomyces albidoflavus* [26]. For this purpose, the biosynthetic gene cluster (BGC) of the compound must be assembled using synthetic genes from the natural plant source, adapted after some modifications (such as codon optimization), which enable its expression in the selected microbial host. 

In the case of eriodictyol, five genes are necessary for its biosynthesis: a tyrosine ammonia-lyase (TAL) converts L-tyrosine to p-coumaric acid (p-CA), which is subsequently activated into coumaroyl-CoA by a 4-coumarate-CoA ligase (4CL). Then, one molecule of coumaroyl-CoA is condensed with three molecules of malonyl-CoA by chalcone synthase (CHS) to give rise to naringenin chalcone, which is then isomerized into naringenin via chalcone isomerase (CHI). Finally, naringenin is converted to eriodictyol by a flavonoid 3′-hydroxylase (F3′H), which is a membrane-bound cytochrome P450 monooxygenase [25,26]. 

The major challenge in the microbial biosynthesis of eriodictyol and other flavonoids is their low production efficiency, normally due to the limited availability of precursors and cofactors [21]. In this respect, many strategies have been adopted in order to increase the malonyl-CoA supply, with numerous examples of metabolic engineering favoring the metabolic fluxes of central carbon metabolism pathways towards malonyl-CoA [21,27,28], including the heterologous expression of *matBC* from *Rhizobium trifolii* to increase cytosolic malonyl-CoA upon exogenous malonate feeding [29,30]. Apart from that, the biosynthesis of hydroxylated flavonoids such as eriodictyol involves another limitation: the low activity associated with plant P450-related enzymes expressed in bacterial hosts [26]. These monooxygenases are membrane-bound enzymes anchored [28] to the endoplasmic reticulum in plant cells, and usually they require an associated P450 reductase coworker. This issue has been addressed in *E. coli* by creating a chimeric protein containing both a P450 hydroxylase and a reductase without their respective membrane-binding regions [21]. 

Among all the aforementioned microorganisms used as microbial cell factories for these polyphenols, *Streptomyces* is the only organism, apart from plants and some fungi, known to be able to produce flavonoids naturally [31,32]. These Gram-positive bacteria are known for their ability to produce a plethora of secondary metabolites, including malonyl-CoA-derived compounds. Currently, there are many metabolic engineering strategies that have been successfully applied to *Streptomyces* to increase malonyl-CoA intracellular levels [33,34,35]. Of note is a strategy exclusive to this microbial host that consists of removing endogenous BGCs that use malonyl-CoA as a precursor as a way of channeling this cytosolic precursor toward the desired heterologously biosynthesized metabolite (e.g., a flavonoid) [36,37]. However, despite all these advantages, no reports have been found of metabolic engineering efforts made in *Streptomyces* in order to increase eriodictyol production. 

In this work, a SEVA-based plasmid collection has been created for synthetic BGC assembly in *Streptomyces* and other actinomycetes using Golden Standard (GS) technology in a plug-and-play manner, as well as for host genomic editing using CRISPR-Cas9 techniques [38]. These tools have been applied for assembling the synthetic eriodictyol BGC into two individual integrative plasmids and for generating an edited version of the *S. albidoflavus* bacterial factory genome via CRISPR-Cas9. This genomic editing involved the replacement of three chromosomal BGCs (encoding malonyl-CoA-derived molecules) by P_ermE*_-*matBC*. Finally, eriodictyol de novo production has been tested in wild-type (WT) and edited strains, validating the new *S. albidoflavus* edited strain as an eriodictyol overproducer. 

## 2. Results

### 2.1. SEVA-Based Plasmid Library Design and Construction

A collection of SEVA modular shuttle vectors for *E. coli* and *Streptomyces* has already been developed by our group [39]. These plasmids are composed of four interchangeable modules: (1) origins of replication for *E. coli* and *Streptomyces*, (2) cargo, (3) antibiotic resistance marker, (4) origins of transfer. Each module is flanked by unique restriction sites for unusual enzymes. Thus, each plasmid or its derived construct can be easily repurposed by exchanging the corresponding module, when necessary, by restriction ligation. However, the SEVA shuttle plasmids developed in this work contain some important extra modifications. The oriT module has been modified in order to include the *traJ* gene to increase conjugation efficiency, and UNS sequences [40] have been added flanking each module to enable plasmid generation by Gibson assembly (GA) of the interchangeable parts. The unique restriction sites between modules have been conserved. In addition, a UNS sequence and a *Nhe*I unique restriction site have been added between the origins of replication for *E. coli* and *Streptomyces* in order to separate them into two different modules. A complete list of the plasmids comprising the library as well as the plasmid derivatives used in this study can be found in Table 1.

The present collection of plasmids is organized into plasmids for *Streptomyces* genome engineering using CRISPR-Cas9 and plasmids for BGC assembly using Golden Standard. CRISPR-Cas9-based genome engineering relies on the ability of the endonuclease Cas9 to cleave a target double-stranded DNA (dsDNA) guided by a synthetic guide RNA (sgRNA). Precise genome editing can be achieved through the introduction of a user-designed editing template including homologous recombination arms [41]. The Golden Standard technique combines the sequential use of different type IIS restriction enzymes, ordered fusion sites, and antibiotic resistance markers in plasmids of different levels to allow the systematic and hierarchical assembly of complete transcription units (TUs) (Level 1) and of multigene constructs, such as BGCs (Level 2), from basic premade standardized modules, such as promoters, rbs, coding sequences, terminators and others (Level 0 parts) [42]. 

**Figure 1:**
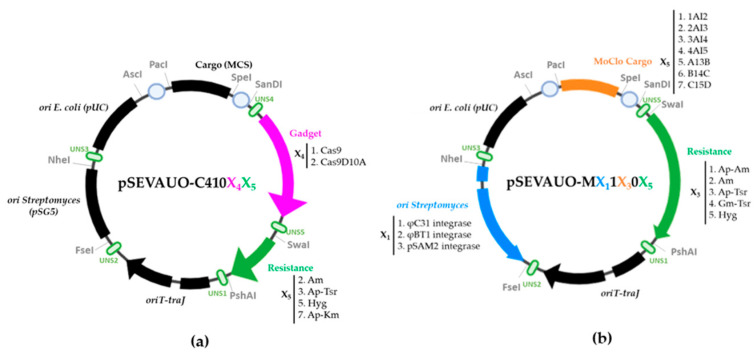
Schematic representation of the SEVA-based plasmid library for (**a**) CRISPR-Cas9-mediated genome edition, and (**b**) Golden Standard assembly of transcriptional units and biosynthetic pathways. Code names: C: CRISPR-Cas9; M: MoClo; X1: ori *Streptomyces*; X2: ori *E. coli*; X3: Cargo; X4: gadget; X5: resistance.

The plasmids for the CRISPR-Cas9-mediated genome edition possess five UNS-flanked modules. Module 1 is flanked by UNS2 (unique nucleotide sequence 2) and UNS3 sequences and harbors the pSG5 origin of replication in *Streptomyces* [43] for easy plasmid curing after the genome edition. Module 2 is flanked by UNS3 and UNS4 sequences and contains pUC as the origin of replication in *E. coli* and a *Pac*I-*Spe*I-flanked multicloning site (MCS) for cloning of the DNA repair template. Module 3 is flanked by UNS4 and UNS5 sequences and contains the CRISPR-Cas9 machinery (cas9-sgRNA) [44,45]. Based on previous studies [46], P_ermE*_ was chosen to lead Cas9 expression. Two versions of Cas9 were generated: the wild-type Cas9, which cuts both DNA strains, and a Cas9 nickase (Cas9D10A), which has one nuclease domain deactivated and therefore only cuts one DNA strand. Module 4 is flanked by UNS5 and UNS1 sequences and consists of the resistance marker, which can be apramycin, ampicillin-thiostrepton, ampicillin-kanamycin or hygromycin. A rational choice of the antibiotic selection marker allows us to carry out CRISPR-Cas9-mediated genome modifications in any strain with Golden Standard plasmids already integrated into their chromosome. Module 5 is flanked by UNS1 and UNS2 sequences and contains the oriT-*traJ* operon (Figure 1). 

The first set of plasmids to be generated were the wild-type Cas9-bearing pSEVAUO-C41012, pSEVAUO-C41013, pSEVAUO-C41017 and pSEVAUO-C41015. They differ in the resistance marker, encoding for apramycin, ampicillin-thioestrepton, ampicillin-kanamycin and hygromycin, respectively. Plasmid pSEVAUO-C41022 contains a D10A mutation in Cas9 for RuvC domain inactivation, leading to Cas9 nickase. Its derivatives, pSEVAUO-C41023 (ampicillin-thiostrepton), pSEVAUO-C41027 (ampicillin-kanamycin) and pSEVAUO-C41025 (hygromycin), allow the use of other antibiotics as selection markers (Supplementary Material, Methods for Plasmids Construction). 

Plasmids for Golden Standard assembly were designed in three levels. Level 0 plasmids harbor basic parts, such as promoters (SP43, SP25, SF14 and ermE* [45,46,47]), a genetic insulator (RBS–RiboJ-SR41 [47]), coding sequences (TAL, 4CL, CHS, CHI and F3′H-CPR) and a terminator (ttsbib [48]). These parts were assembled in TUs using Level 1 plasmids as destination vectors, and from these into basic circuits, such as BGCs, when assembled into Level 2 plasmids. Level 0 Golden Standard plasmids are ampicillin-resistant (normally pSEVA181 [48]). 

Level 1 and Level 2 Golden Standard plasmids possess four UNS-flanked modules. Module 1 is flanked by UNS2 and UNS3 sequences, and it harbors an integrase and its corresponding attP site for stable integration of the plasmid in the *Streptomyces* chromosome. There are three optional integrases: φC31, φBT1 and pSAM2 [49,50,51,52]. Module 2 is flanked by UNS3 and UNS5 sequences and consists of the pUC origin of replication for *E. coli* and a *Pac*I-*Spe*I-flanked Golden Standard cargo region. The configuration of each Golden Standard cargo region can be found in the literature [38]. Module 3 is flanked by UNS5 and UNS1 sequences and contains the antibiotic resistance marker. There are five different resistance marker cassettes: apramycin, ampicillin-thiostrepton, gentamicin-thiostrepton, apramycin-ampicillin and hygromycin. These resistance markers have been combined with integrases and Golden Standard cargo regions to enable the selection of assembled plasmids in Golden Standard reactions as well as their integration into the *Streptomyces* chromosome. Module 4 is flanked by UNS1 and UNS2 sequences and harbors oriT and traJ (Figure 1). A set of Level 1 and Level 2 Golden Standard plasmids displaying resistance to apramycin or ampicillin and to be integrated into the φC31 site were developed, including pSEVAUO-M11101 (cargo 19[g1]), pSEVAUO-M11201 (cargo 19[g2]), pSEVAUO-M11301 (cargo 19[g3]), pSEVAUO-M11401 (cargo 19[g4]), pSEVAUO-M11501 (cargo 19[gA]), pSEVAUO-M11601 (cargo 19[gB]) and pSEVAUO-M11701 (cargo 19[gC]). Then, a set of plasmids to be integrated into φBT1 site were generated, including pSEVAUO-M21102 (GS cargo 19[g1]), pSEVAUO-M21202 (GS cargo 19[g2]), pSEVAUO-M21302 (GS cargo 19[g3]) and pSEVAUO-M21402 (GS cargo 19[g4]). The same process was applied to the generation of Level 2 φBT1 Golden Standard plasmids pSEVAUO-M21503 (GS cargo 19[gA]), pSEVAUO-M21603 (GS cargo 19[gB]) and pSEVAUO-M21703 (GS cargo 19[gC]) but encoding resistance to ampicillin-thiostrepton instead of apramycin. 

Level 1 φBT1-apramycin Golden Standard plasmids could not be integrated into Streptomyces chromosomes together with φC31-ampicillin-apramycin plasmids, as they also possess apramycin as a selector marker. Therefore, a series of Level 1 and Level 2 φBT1 plasmids with resistance to gentamicin-thiostrepton were built. Gentamicin works as a selector marker for both Level 1 and Level 2 Golden Standard reactions in *E. coli*, whilst selection in *Streptomyces* is carried out with thiostrepton, allowing the right integration even in the presence of other Golden Standard integrative plasmids. Level 1 φBT1-gentamicin-thiostrepton plasmids, pSEVAUO-M21104 (cargo 1AI2), pSEVAUO-M21204 (cargo 2AI3), pSEVAUO-M21304 (cargo 3AI4) and pSEVAUO-M21404 (cargo 4AI5), were generated individually by 4-fragment GA but using fragments already containing the corresponding Golden Standard cargos. Level 2 φBT1-gentamicin-thiostrepton plasmids pSEVAUO-M21504 (cargo A13B), pSEVAUO-M21604 (cargo B14C) and pSEVAUO-M21704 (cargo C15D) were generated by restriction ligation of the corresponding cargos into pSEVAUO-M21404. A third series of plasmids containing the pSAM2 integrase and encoding resistance to hygromycin was developed. This antibiotic serves as a selector in both *E. coli* and *Streptomyces*. The first plasmid of this series to be generated was pSEVAUO-M31705, which was assembled by a 4-fragment Gibson assembly using a fragment already containing cargo C15D. Plasmids pSEVAUO-M31105 (cargo 1AI2), pSEVAUO-M31205 (cargo 2AI3), pSEVAUO-M31305 (cargo 3AI4), pSEVAUO-M31405 (cargo 4AI5), pSEVAUO-M31505 (cargo A13B) and pSEVAUO-M31605 (cargo B14C) were generated by cloning the corresponding cargos into pSEVAUO-M31705. 

### 2.2. Eriodictyol Heterologous Biosynthesis in S. albidoflavus WT

De novo eriodictyol (ERI) biosynthesis by *S. albidoflavus* was already achieved by our group, but titers were low [26]. This was associated with poor activity of the enzyme F3′H (flavonoid 3’-hydroxylase), which catalyzes the hydroxylation of naringenin at ring B to give rise to eriodictyol. Heterologous expression of this enzyme in prokaryotic systems is normally challenging due to its poor solubility and cofactor incorporation [21]. Therefore, the F3′H sequence was redesigned in order to encode a chimeric protein comprising a truncated F3′H fused to a truncated cytochrome P450 reductase (CPR), both proteins from *Arabidopsis thaliana*.

**Figure 2:**
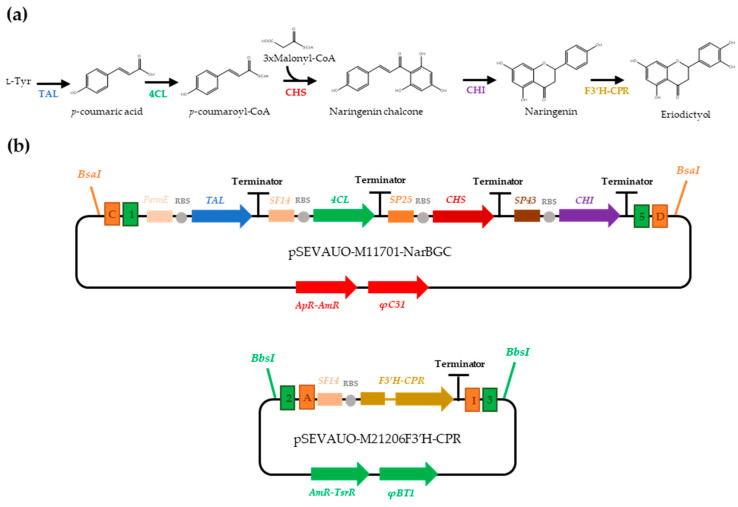
Eriodictyol biosynthesis. (**a**) Biosynthetic pathway, (**b**) MoClo plasmids bearing naringenin biosynthetic gene cluster (pSEVAUO-M11701-NAR-BGC) and F3′H-CPR transcriptional unit (pSEVAUO-M21206-F3′H-CPR) for naringenin conversion to eriodictyol.

First, the plasmid pSEVAUO-M11701-NAR-BGC was generated. This is a φC31 integrative Level 2 Golden Standard plasmid harboring the four genes necessary for naringenin biosynthesis (TAL, 4CL, CHS and CHI), each of them with their own promoter, RiboJ–RBS and terminator. Promoters of increasing strength were assigned to each CDS according to the order in which their encoded enzyme acted on the naringenin (NAR) biosynthesis pathway (Figure 2). This plasmid was integrated into the φC31 chromosomal site of *S. albidoflavus* WT by protoplast transformation, giving rise to the strain *S. albidoflavus* WT-NAR. Then, pSEVAUO-M21206-F3′H-CPR, a φBT1 integrative Level 1 Golden Standard plasmid was assembled. This harbors the gene encoding the F3′H-CPR chimaera under the control of the SF14 promoter (Figure 2). Integration of this plasmid into the φBT1 chromosomal site of the strain *S. albidoflavus* WT-NAR gave rise to the strain *S. albidoflavus* WT-ERI.

**Figure 3:**
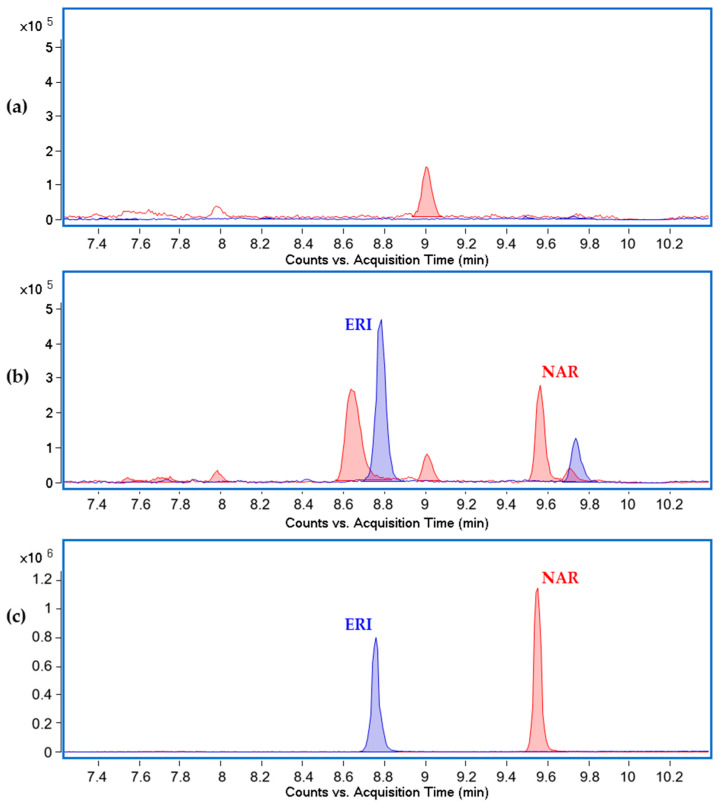
HPLC–HRESIMS chromatograms of (**a**) *S. albidoflavus* WT, (**b**) *S. albidoflavus* WT-ERI and (**c**) pure flavonoid standards; filtered by naringenin at *m*/*z* ion = 271.0612 (red), and eriodictyol ion at *m*/*z* ion = 287.0561 (blue), both in negative mode. ERI: eriodictyol, NAR: naringenin.

The strains *S. albidoflavus* WT-NAR, WT-ERI and wild-type (as a negative control) were cultured in an R5A liquid medium. De novo production of naringenin by the strains *S. albidoflavus* WT-NAR and *S. albidoflavus* WT-ERI and eriodictyol production by the strain *S. albidoflavus* WT-ERI were validated by comparison of differential peaks with pure standards in HPLC–HRESIMS (Figure 3). 

### 2.3. Comparison of Conjugation and Genome Editing Efficiency between Wild-Type Cas9 and Cas9 Nickase

Cas9 expression has been described as toxic in *Actinobacteria*, but its toxic effects are circumvented by the proper selection of the promoter driving its expression [53]. Alternatively, a Cas9D10A nickase, which consists of a RuvC nuclease-defective Cas9, which introduces only a single-strand nick to the targeted DNA, has been adopted to overcome the same limitation in *Clostridium cellulyticum* [52]. Therefore, we have created a plasmid library with both the wild-type Cas9 and the Cas9 nickase and have tested their effectiveness in *S. albidoflavus*.

**Figure 4:**
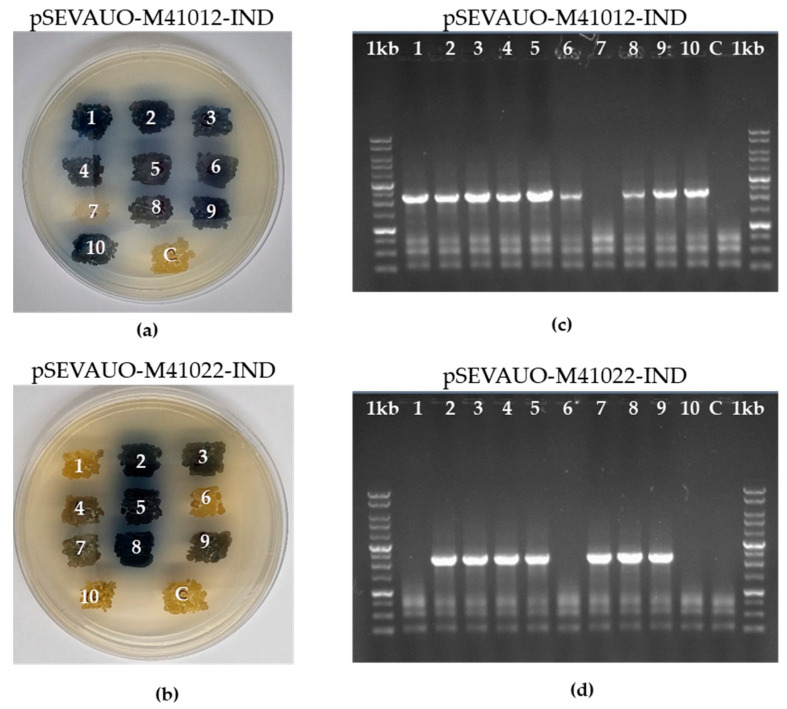
Comparison of the editing efficiency of wild-type Cas9 and Cas9 nickase by indigoidine gene cluster activation. Plates showing 10 colonies from conjugations with (**a**) pSEVAUO-M41012-IND (wild-type Cas9) and (**b**) pSEVAUO-M41022-IND (Cas9 nickase) as well as PCR verification of exconjugants from (**c**) pSEVAUO-M41012-IND (wild-type Cas9) and (**d**) pSEVAUO-M41022-IND (Cas9 nickase) are displayed. A negative control (C) was included in each experiment. 1kb: commercial DNA ladder.

The conjugation and editing efficiency of both the wild-type Cas9 and the Cas9 nickase have been tested by activation of the *S. albidoflavus* endogenous BGC encoding for the blue compound indigoidine (IND). This BGC remains naturally silent in *S. albidoflavus*. However, when the constitutive promoter P_ermE*_ is placed upstream of the first gene of the BGC, its expression is activated and colonies turn blue [53], enabling visual identification of recombinant strains. For this purpose, pSEVAUO-C41012-IND and pSEVAUO-C41022-IND have been constructed and separately introduced into *S. albidoflavus* WT by conjugation. The conjugation efficiency was measured as the number of colonies growing on a plate, and this was calculated as the mean of three independent experiments. The conjugation efficiency obtained with wild-type Cas9 was 195 ± 38 CFU, in contrast to 459 ± 78 CFU obtained with Cas9 nickase. The editing efficiency was calculated as the percentage of edited colonies out of 10 exconjugants selected from each conjugation and verified by PCR with the primers P_ermE_*-fw (annealing on P_ermE*_) and P_ermE*_-IND-check-rev (annealing on the chromosome outside the fragment used as part of the DNA repair template) (Supplementary Table S3). In this case, the recombination efficiency was higher when the wild-type Cas9 was used (90%) compared to the Cas9 nickase (70%) (Figure 4). In light of these results, the plasmids bearing wild-type Cas9 were selected for subsequent experiments in *S. albidoflavus*.

### 2.4. Generation of the S. albidoflavus UO-FLAV-002 Edited Bacterial Factory

The CRISPR-Cas9-bearing plasmids were used to generate the *S. albidoflavus* UO-FLAV-002 bacterial factory in two steps: (1) the removal of chromosomal pseB4, a highly active pseudo-attB site for φC31 alternative integration; (2) the replacement of a series of native BGCs by the construction of P_ermE*_-matBC, in order to increase malonyl-CoA levels.

To direct the specific integration of φC31 integrative plasmids to only one site, the pseB4 pseudo-attB site was removed from the *S. albidoflavus* chromosome to ensure uniformity throughout the flavonoid production experiments. For this purpose, plasmid pSEVAUO-C41012-pSEB4 was introduced into the *S. albidoflavus* J1074 WT strain for pSEB4 replacement by a non-coding UNS8 sequence through CRISPR-Cas9-mediated recombination. The correct genome edition of the derived strain, *S. albidoflavus* UO-FLAV-001, was verified by PCR amplification (Supplementary Figure S1) and the sequencing of the obtained PCR products.

*S. albidoflavus* UO-FLAV-001 was further genetically modified to increase malonyl-CoA availability for flavonoid production. Two strategies were adopted for this purpose. Three biosynthetic gene clusters (BGC) encoding malonyl-CoA-derived metabolites, such as antimycins, candicidins and a predicted non-ribosomal peptide-polyketide, were removed from the *S. albidoflavus* chromosome and the *matBC* genes from *R. trifolii* were integrated into the chromosome under the control of the ermE* promoter. The operon *matBC* improves intracellular malonyl-CoA upon exogenous malonate addition to the culture, as MatC imports malonate into the cell and MatB converts this malonate into malonyl-CoA [30]. The final *S. albidoflavus* UO-FLAV-002 edited bacterial factory was therefore generated by a CRISPR-Cas9-mediated replacement of a 241,776 bp fragment (CP004370; DNA region from 6,576,725 to 6,818,501 bp) comprising the three mentioned BGCs (Cluster 22 henceforth) by P_ermE*_-matBC. To this end, plasmid pSEVAUO-C41012-C22-*matBC* was introduced into *S. albidoflavus* UO-FLAV-001 by conjugation. The correct genome edition was checked by PCR amplification (Supplementary Figure S2) and the sequencing of the PCR products. The successful blockage of antimycin biosynthesis was demonstrated by culturing the *S. albidoflavus* UO-FLAV-002 edited strain and the wild-type strain (as a control) in R5A over 120 h and further analyses of the extracts by HPLC–HRESIMS (Supplementary Figure S3). Candicidins were not detected in this edited strain extract.

### 2.5. Eriodictyol Heterologous Biosynthesis in S. albidoflavus UO-FLAV-002

The performance of the *S. albidoflavus* UO-FLAV-002 edited strain as a flavonoid-overproducing bacterial factory was investigated by testing its eriodictyol heterologous production. First, the plasmid pSEVAUO-M11701-NAR-BGC was integrated into the φC31 site of *S. albidoflavus* UO-FLAV-002 by protoplast transformation to generate the *S. albidoflavus* UO-FLAV-002-NAR strain. This strain was further modified by the integration of the plasmid pSEVAUO-M21206-F3′H-CPR into its φBT1 chromosomal site, giving rise to *S. albidoflavus* UO-FLAV-002-ERI. 

Then, the *S. albidoflavus* WT-ERI and *S. albidoflavus* UO-FLAV-002-ERI strains were cultured in R5A in order to test the impact on eriodictyol production after removing the mentioned three malonate-consuming BGCs. Additionally, the *S. albidoflavus* UO-FLAV-002-ERI strain was cultured in R5A with malonate at 20 mM in order to test the effect of the presence of MatBC on eriodictyol production. 

**Figure 5:**
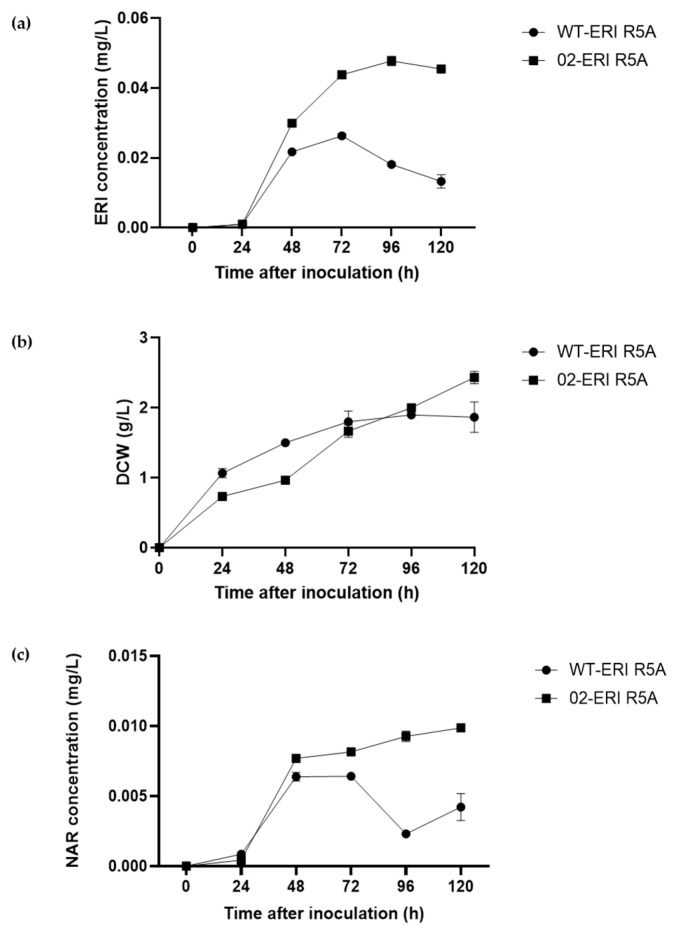
*S. albidoflavus* WT-ERI and *S. albidoflavus* UO-FLAV-002-ERI cultured in R5A. Time courses of eriodictyol production (**a**), biomass (**b**) and naringenin production (**c**).

Both parameters, the eriodictyol production and the biomass, were monitored every 24 h over a cultivation period of 120 h. Indeed, the *S. albidoflavus* UO-FLAV-002-ERI strain produced more eriodictyol than the WT strain, with a maximum of 0.06 mg/L yield 96 h after the inoculation. This constitutes a 3.44-fold compound increase over the maximum level produced by the WT strain (0.033 mg/L, 72 h after the inoculation) (Figure 5a). In terms of growth, the *S. albidoflavus* UO-FLAV-002-ERI strain grew slower than the WT strain at early time points but reached equal biomass at the end of the cultivation (Figure 5b). Both strains were also tested for naringenin production, and the *S. albidoflavus* UO-FLAV-002-ERI strain showed a 2.33-fold increase in its production (Figure 5c). Unfortunately, no major differences were observed between *S. albidoflavus* UO-FLAV-002-ERI cultured with and without malonate. This result is expected as malonyl-CoA was no longer a limiting factor to eriodictyol production in the given cultivation conditions.

## 3. Discussion

As in many other actinomycete bacteria, the *S. albidoflavus* chromosome contains a plethora of BGCs, which drive processes associated with the biosynthesis of a variety of native secondary metabolites, such as polyketides, terpenoids, siderophores, bacteriocins, and non-ribosomal peptides (NRPs) [53]. In the case of *S. albidoflavus*, a peripheral region of its chromosome (CP004370; 6,841,649 bp) [54], located between 6,576,725 and 6,818,501 bp, contains the gene clusters involved in the biosynthesis of candicidins, antimycins and an unknown hybrid polyketide–NRP compound [53], as predicted by the ANTISMASH software [55,56]. The biosynthetic pathways associated with these secondary metabolites share the use of high quantities of malonyl-CoA as one of their building blocks. For example, candicidins are antifungal type I polyketides using 14 malonyl-CoA building blocks per molecule (rendering acetate moieties in the final polyene backbone) [57]. In addition, antimycins are antifungal, antitumor and piscicidal depsipeptides generated by a hybrid polyketide and NRPS complex enzymatic machinery that uses, as biosynthetic precursors, L-Trp (to generate the starter unit 3-aminosalicylate) and diverse amino acids and carboxylic acids as extension units [58]. These include pyruvate, a precursor of acetyl-CoA (via pyruvate dehydrogenase) and therefore of malonyl-CoA biosynthesis [59,60,61]. The structure of the secondary metabolite generated by the third gene cluster in this chromosomal region (coding for a hybrid polyketide–NRP compound) is unknown, but this gene cluster was deleted as it may also require the consumption of malonyl-CoA (as this is the most common building block in polyketide moieties).

To achieve higher eriodictyol production yields in this bacterial factory, a deletion (using CRISPR-Cas9) of the previously mentioned chromosomal region was carried out, comprising about 242 kb of chromosomal DNA. The objective here was to divert the unused cytoplasmic malonyl-CoA in the new edited strain (*S. albidoflavus* UO-FLAV002) towards the biosynthesis of this flavanone: each molecule of eriodictyol uses three malonyl-CoA building blocks and one p-coumaroyl-CoA as precursors. In addition, a further step towards the enhancement of malonyl-CoA cytoplasmic pools was the insertion of the plant *matBC* genes from *R. trifolii* (under a constitutive promoter) during this chromosomal editing event. These two genes code for a dicarboxylate membrane transporter in charge of introducing malonate from the extracellular medium (MatC) and a malonyl-CoA synthetase, which intracellularly converts the imported malonate into the malonyl-CoA building block [30]. As a result of this genetic replacement in the *S. albidoflavus* chromosome, the strain *S. albidoflavus* UO-FLAV-002 was generated and tested to produce antimycins and candicidins by HPLC–HRESIMS. Both secondary metabolites were absent in the edited strain extracts, but a large quantity of *m/z* peaks corresponding to the structural diversity of antimycins was easily observable in the WT strain extracts (Supplementary Figure S3). 

Additionally, in this work, the integration systems from the temperate phages φC31 and φBT1 and from the conjugative element pSAM2 have been used to allow the stable integration of the Golden Standard plasmids developed here into specific loci at the *Streptomyces* chromosome. Although they have a preferred chromosomal site of integration, sometimes these integrases might carry out the recombination event at alternative pseudo-*attB* sites in the target chromosome, but at a far lower efficiency [62]. In this sense, a highly active pseudo-*attB* site for the φC31 integrase has been identified in the *S. albidoflavus* chromosome. This pseudo-*attB* site, named pseB4, comprises a 50 nt sequence (CP004370; DNA region from 3,168,410 to 3,168,459 bp) located in the intergenic region between the XNR_2791 and XNR_2792 genes, and it exhibits a similar integration efficiency as the native *attB* [63]. This pseudo-*attB* site was therefore deleted (using CRISPR-Cas9 edition) to maintain a unique integration site along the bacterial factory chromosome for the φC31-derived integrative plasmid vectors created, avoiding transcriptional level differences for the eriodictyol biosynthetic genes used (eventually derived from different chromosomal integration sites with variable transcription level capabilities) [64], as well as preventing eventual duplication of the heterologous gene cluster in the chromosome. 

With the pseudo-*attB* site deleted from the bacterial chromosome, as well as the three mentioned biosynthetic gene clusters, the introduction of all the synthetic genes for the heterologous production of the flavanone eriodictyol (and its precursor naringenin) was achieved both in the WT and in the UO-FLAV-002 edited bacterial factory. The four genes necessary for naringenin biosynthesis were integrated into the canonical φC31 *attB* site, and the gene coding for the F3′H-CPR chimaera was integrated into the φBT1 *attB* site. With the modifications carried out in the edited bacterial factory, total naringenin production titers achieved a 2.33 increase with respect to the WT strain, and total eriodictyol yields reached a 3.44 rise with respect to the WT conditions; therefore, validating the fact that saving biosynthetic building blocks (in this case malonyl-CoA), due to the deletion of these three native gene clusters, is a reliable approach for enhancing final flavonoids heterologous production titers in this actinomycete. However, the same production experiments for eriodictyol biosynthesis, but adding 20 mM malonate to the R5A culture media, did not render an increase in the final production yields, probably indicating that, after deleting these three native gene clusters in *S. albidoflavus*, the intracellular levels of malonyl-CoA are no longer a limiting factor for the heterologous production of these flavonoids. This also indicates that, in the WT strain (with full native machinery for the biosynthesis of secondary metabolites such as antimycins and others), the addition of malonate to the culture media does not generate a net increase in the heterologously produced flavonoids. 

## 4. Materials and Methods

### 4.1. Bacterial Strains, Plasmids and Culture Conditions

All plasmids and strains used in this study are listed in Tables S1 and S2. *E. coli* strains were grown at 37 °C in Tryptic Soy Broth (TSB, VWR, Barcelona, Spain) or on TSB agar plates. *S. albidoflavus* J1074 (formerly denominated *S. albus* J1074) and derivatives were grown at 30 °C in Yeast Extract Malt Extract 17% (*w*/*v*) sucrose (YEME) [65] for protoplast preparation, medium A [66] supplemented with MgCl_2_ 10 mM for conjugation, or Bennett medium [65] for sporulation. Media were supplemented with the corresponding antibiotics (ampicillin 100 µg/mL, apramycin 50 µg/mL, kanamycin 50 µg/mL for *E. coli* and 200 µg/mL for *S. albidoflavus*, hygromycin 100 µg/mL, nalidixic acid 50 µg/mL, gentamicin 50 µg/mL, thiostrepton 50 µg/mL on solid and 5 µg/mL in liquid medium) and reagents (X-Gal 40 µg/mL), when necessary.

For flavonoid production, *S. albidoflavus* spores were quantified, and 10^6^ CFU/mL were inoculated into R5A medium [66]. Cultures were incubated at 30 °C and 250 rpm. 

### 4.2. Reagents and Biochemicals

All solvents used for solid phase extraction and HPLC–HRESIMS analysis were LC-MS grade from either Sigma-Aldrich (Madrid, Spain) or VWR (Barcelona, Spain).

Authentic standards of naringenin and eriodictyol for HPLC–HRESIMS quantification and molecule identification were provided by Extrasynthese (Genay, France).

### 4.3. Synthetic DNA and Enzymes

Recombinant DNA techniques were performed following standard protocols [67]. Restriction enzymes and T4 DNA ligase were purchased from Thermo Scientific (Madrid, Spain), NEBuilder® HiFi DNA Assembly Master Mix from New England BioLabs (Thermo Scientific, Madrid, Spain), Terra PCR Direct polymerase from Takara (DISMED, Gijón, Spain), and Herculase II Fusion DNA Polymerase from Agilent (Madrid, Spain). Synthetic genes outsourced from Explora Biotech (Venice, Italy) were received and cloned into *Pac*I-*Spe*I sites of pSEVA181. All gene sequences were modified to include silent point mutations to remove restriction sites incompatible with Golden Standard and SEVA architecture and to meet the criteria to allow chemical synthesis when necessary [68]. For the antibiotic resistance cassettes, *San*DI-UNS5-*Swa*I and *Psh*AI-UNS1 sequences were added as flanking regions: pSEVA181Tsr contains the thiostrepton resistance cassette from pGM1190; pSEVA181Hyg contains the hygromycin resistance cassette from pOSV805. For the site-specific integrases, UNS3-*Nhe*I and *Fse*I-UNS2 sequences were added as flanking regions: pSEVA181BT1int contains the φBT1 integrase and its *attP* site from pOSV805; pSEVA181pSAM2 contains the pSAM2 integrase and its *attP* site from pOSV807. For the Level 0 Golden Standard plasmids harboring promoter regions, *Bsa*I-GGAG (Fusion site A) and TACT (Fusion site B)-*Bsa*I sequences were added as flanking regions: pSEVA181P_ermE_*, pSEVA181SF14, pSEVA181SP25 and pSEVA181SP43 contain the P_ermE*_, SF14, SP25 and SP43 promoter sequences, respectively [69]. Plasmid pSEVA181RiboJ-RBS contains the insulator RiboJ [70] followed by the RBS sequence SR41 [49,69] flanked by *Bsa*I-TACT (Fusion site B) and AATG (Fusion site D)-*Bsa*I. For the Level 0 Golden Standard plasmids harboring coding sequences, *Bsa*I-AATG (Fusion site D) and GCTT (Fusion site G)-*Bsa*I were added as flanking regions: pSEVA181CHS contains a codon-optimized CHS gene from *Glycine max* (GenBank accession no. LT629807.1); pSEVA181CHI contains a codon-optimized CHI gene from *Glycine max* (GenBank accession no. LT629808.1); and pSEVA181F3H-CPR contains a codon-optimized F3′H-CPR chimaera from *Arabidopsis thaliana* (accession number: OQ674225). This fusion protein was designed as described elsewhere [21]: the sequence of a previously codon-optimized F3′H (GenBank accession no. LT629809.1) was modified in order to remove the first 21 amino acids corresponding to the transmembrane region. An ATG codon was added at 5′ and a linker Gly-Ser-Thr at 3′. Consecutively, the nucleotide sequence encoding a truncated NADPH-cytochrome P450 reductase (CPR) was added. This sequence was obtained from the protein sequence of isoform 1 of gen ATR2 from *A. thaliana* (UniProt accession no. Q9SUM3-1) and, after codon optimization, nucleotides encoding the first 72 amino acids corresponding to the transmembrane region were removed. Plasmid pIDTSMARTttsbib was outsourced from IDT Technologies (Leuven, Belgium) and contains the terminator *ttsbib* [71] flanked by *Bsa*I-GCTT (Fusion site G) and CGCT (Fusion site I)-*Bsa*I. Plasmid p*matBC* was de novo designed and contains codon-optimized *matBC* genes from *R. trifolii*, each preceded by an RBS sequence (Accession number: OQ659402). A 499 bp gBlock for cloning two protospacer sequences targeting Cluster 1 was designed following the corresponding protocol [44] and outsourced from IDT Technologies (Leuven, Belgium).

### 4.4. Construction of Plasmids

A detailed description of each plasmid construction can be found in the Supplementary Material (Methods for Plasmids Construction). Sequence of all primers used can be found in Supplementary Table S3. Gibson Isothermal Assembly (GA) was performed with NEBuilder® HiFi DNA Assembly Master Mix (New England BioLabs) (Thermo Scientific, Madrid, Spain), following the manufacturer’s instructions.Golden Standard reactions were set up according to CIDAR MoClo [44], followed by blue–white screening by adding X-Gal to the medium. Protospacer design, annealing and cloning were performed as described for pCRISPomyces-2 [44]. Correct protospacer insertion was verified by sequencing with primer “Prot seq rev”. Correct assembly of all constructs was first checked by restriction digestion and then confirmed by sequencing. The GenBank accession numbers for these plasmids are OQ696801 to OQ696836. Supplementary Table S1 contains information on all the plasmids used in this work. All these vectors’ data will be available from the Golden Standard database after manuscript publication and can be requested directly from the SEVA siblings repository (http://sysbiol.cnb.csic.es/GoldenStandard/database.php, accessed on 1 May 2023). 

**Figure 6:**
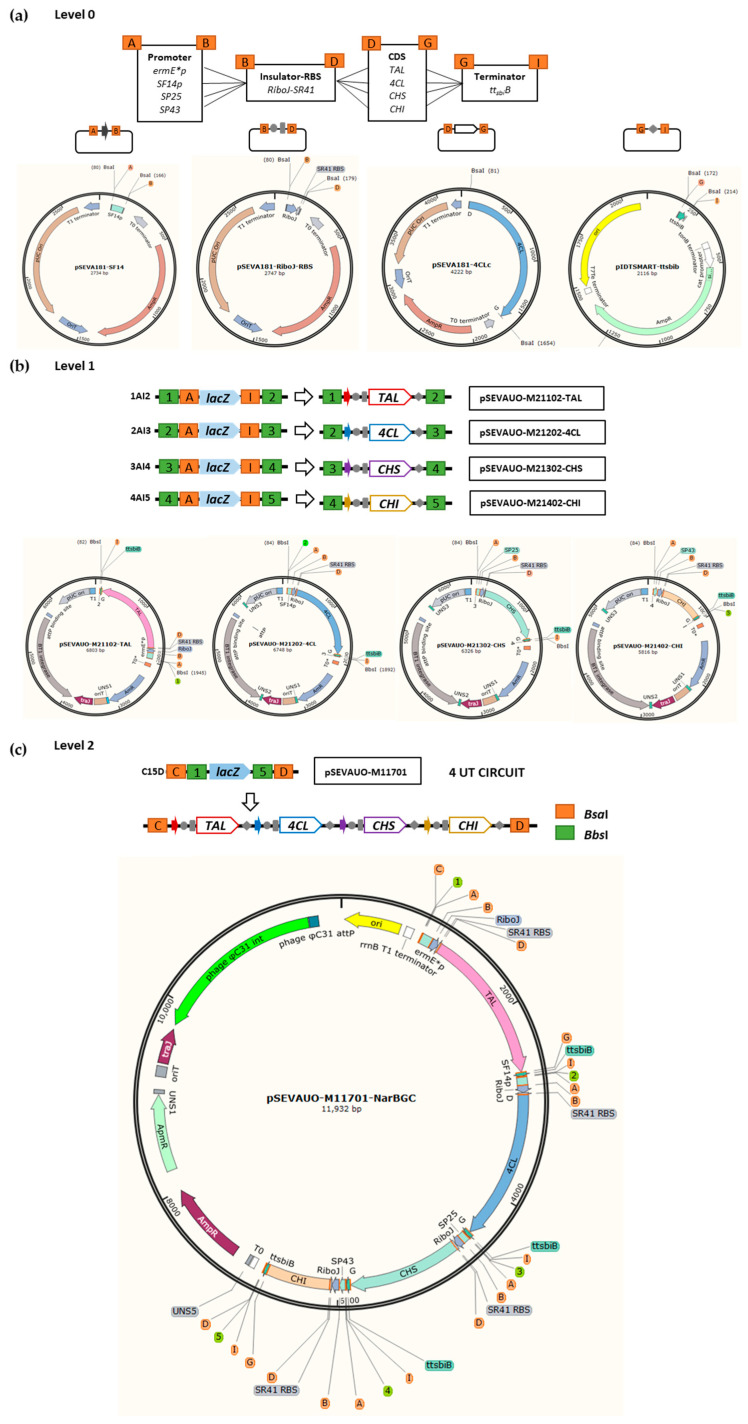
Schema showing the steps needed to generate a level 2 Golden Standard plasmid, designed to direct the biosynthesis of naringenin. (**a**) The different level 0 plasmids containing the structural parts (promoters, rbs, CDSs, terminator). (**b**) The four necessary level 1 plasmids showing the four different gene constructions. (**c**) The final level 2 plasmid generated.

Figure 6 shows a basic schema with an example of the construction of a level 2 vector from the different parts depicted in four level 1 vectors (constructed on the basis of the sixteen possible units of level 0 vectors) (Figure 6).

### 4.5. Strain Generation

Exogenous DNA was introduced into *S. albidoflavus* by either protoplast transformation or intergeneric conjugation [67]. Description of each strain generated by CRISPR-Cas9 and further verification can be found in Supplementary Figures S1 and S2, as well as information about the primers used for PCR amplification (Supplementary Table S3). Strains containing integrative plasmids were selected based on their resistance to the corresponding antibiotics. For the strains generated by CRISPR-Cas9 edition, colonies showing resistance to the corresponding antibiotics were re-streaked for colony isolation. Then, spores from an isolated colony were subjected to PCR verification with Terra polymerase to check both genome edition and contamination with DNA from the non-edited strain. When the positive recombinant colonies showed a mixture with non-edited DNA, the spores were filtered and spread on a plate to obtain isolated colonies with unique genotypes and checked again by PCR. Plasmid curing was achieved with consecutive passages without antibiotics and phenotypically checked by their loss of resistance to that antibiotic. Supplementary Table S2 contains information on all the strains used in this work.

### 4.6. Flavonoid Extraction and HPLC–HRESIMS Analyses 

The recovery of flavonoids from the recombinant strains developed in this study was achieved by organic extraction with acetone (cellular pellet) and ethyl acetate (culture supernatant). Briefly, 1 mL of culture medium was centrifuged at 10,000 rpm for 2 min, and the biomass as well as the supernatant were extracted separately. First, an equal volume of acetone was added to the pellet to facilitate the breaking of the mycelium, and then a second extraction was performed with an equal volume of ethyl acetate. For the supernatant, two extractions were performed with the same volume of ethyl acetate. In all cases, the extractions were subjected to vortex cycles to ensure contact with the solvent. Finally, the organic phases were dried sequentially under vacuum, and the residues were resuspended in 100 μL DMSO/MeOH 1:1 (*v*/*v*) for further analysis.

Analysis of flavonoids was performed using HPLC–HRESIMS. Separation was performed in a UPLC system (Dionex Ultimate 3000, ThermoScientific, Madrid, Spain) equipped with an analytical RP-18 HPLC column (50 × 2.1 mm, Zorbax^®^ Eclipse Plus, 1.8 μm, Agilent Technologies, Madrid, Spain) as previously described [71]. The obtained base peak chromatograms (BPCs) were extracted for the deprotonated ions of a set of flavonoids with a mass error range of 0.005 milli mass units (mmu) and the obtained extracted ion chromatograms (EICs) were compared with authentic commercial standards. When needed, flavonoids were quantified by comparing the peak area with that of a known amount of an authentic compound through a calibration curve. The production titers are given in mg/L, and the mean value was calculated from three biological replicates. 

## Figures and Tables

**Table 1:** Bifunctional shuttle vectors and derivatives generated in this study for bacterial factory genome edition as well as for the assembly of biosynthetic gene clusters. This table includes the plasmid derivatives for the CRISPR-Cas9-mediated removal of the pSEB4 chromosomal alternative integration site and for the replacement of BGC22 by P_ermE*_-matBC, as well as the Golden Standard plasmid vectors for naringenin and eriodictyol biosynthesis. RT: repair template.

Name	Origin *Streptomyces*	Origin *E. coli*	Cargo	Gadget	Resistance	Accession Number
pSEVAUO-C41012	pSG5	pUC	MCS	Cas9	Am	OQ696801
pSEVAUO-C41013	pSG5	pUC	MCS	Cas9	Ap-Tsr	OQ696802
pSEVAUO-C41017	pSG5	pUC	MCS	Cas9	Ap-Km	OQ696803
pSEVAUO-C41015	pSG5	pUC	MCS	Cas9	Hyg	OQ696804
pSEVAUO-C41022	pSG5	pUC	MCS	Cas9D10A	Am	OQ696805
pSEVAUO-C41023	pSG5	pUC	MCS	Cas9D10A	Ap-Tsr	OQ696806
pSEVAUO-C41027	pSG5	pUC	MCS	Cas9D10A	Ap-Km	OQ696807
pSEVAUO-C41025	pSG5	pUC	MCS	Cas9D10A	Hyg	OQ696808
pSEVAUO-M21102	φBT1	pUC	1AI2	-	Am	OQ696809
pSEVAUO-M21202	φBT1	pUC	2AI3	-	Am	OQ696810
pSEVAUO-M21302	φBT1	pUC	3AI4	-	Am	OQ696811
pSEVAUO-M21402	φBT1	pUC	4AI5	-	Am	OQ696812
pSEVAUO-M21503	φBT1	pUC	A13B	-	Ap-Tsr	OQ696813
pSEVAUO-M21603	φBT1	pUC	B14C	-	Ap-Tsr	OQ696814
pSEVAUO-M21703	φBT1	pUC	C15D	-	Ap-Tsr	OQ696815
pSEVAUO-M21104	φBT1	pUC	1AI2	-	Gm-Tsr	OQ696816
pSEVAUO-M21204	φBT1	pUC	2AI3	-	Gm-Tsr	OQ696817
pSEVAUO-M21304	φBT1	pUC	3AI4	-	Gm-Tsr	OQ696818
pSEVAUO-M21404	φBT1	pUC	4AI5	-	Gm-Tsr	OQ696819
pSEVAUO-M21504	φBT1	pUC	A13B	-	Gm-Tsr	OQ696820
pSEVAUO-M21604	φBT1	pUC	B14C	-	Gm-Tsr	OQ696821
pSEVAUO-M21704	φBT1	pUC	C15D	-	Gm-Tsr	OQ696822
pSEVAUO-M11101	φC31	pUC	1AI2	-	Ap-Am	OQ696823
pSEVAUO-M11201	φC31	pUC	2AI3	-	Ap-Am	OQ696824
pSEVAUO-M11301	φC31	pUC	3AI4	-	Ap-Am	OQ696825
pSEVAUO-M11401	φC31	pUC	4AI5	-	Ap-Am	OQ696826
pSEVAUO-M11501	φC31	pUC	A13B	-	Ap-Am	OQ696827
pSEVAUO-M11601	φC31	pUC	B14C	-	Ap-Am	OQ696828
pSEVAUO-M11701	φC31	pUC	C15D	-	Ap-Am	OQ696829
pSEVAUO-M31105	pSAM2	pUC	1AI2	-	Hyg	OQ696830
pSEVAUO-M31205	pSAM2	pUC	2AI3	-	Hyg	OQ696831
pSEVAUO-M31305	pSAM2	pUC	3AI4	-	Hyg	OQ696832
pSEVAUO-M31405	pSAM2	pUC	4AI5	-	Hyg	OQ696833
pSEVAUO-M31505	pSAM2	pUC	A13B	-	Hyg	OQ696834
pSEVAUO-M31605	pSAM2	pUC	B14C	-	Hyg	OQ696835
pSEVAUO-M31705	pSAM2	pUC	C15D	-	Hyg	OQ696836
pSEVAUO-C41012-Ind	pSG5	pUC	P_ermE__*_-Ind DNA RT	Cas9-Ind Prot	Am	This work
pSEVAUO-C41022-Ind	pSG5	pUC	P_ermE__*_-Ind DNA RT	Cas9D10A-Ind Prot	Am	This work
pSEVAUO-C41012-pSEB4	pSG5	pUC	pSEB4::UNS8 DNA RT	Cas9-pSEB4 Prot	Am	This work
pSEVAUO-C41012-C1-matBC	pSG5	pUC	C1::P_ermE__*_-*matBC* DNA RT	Cas9-C1 Prot1-Prot2	Am	OQ659402
pSEVAUO-M21102-TAL	φBT1	pUC	P_ermE__*_-*TAL*	-	Am	This work
pSEVAUO-M21202-4CL	φBT1	pUC	*SF14*-4CL	-	Am	This work
pSEVAUO-M21302-CHS	φBT1	pUC	*SP25-CHS*	-	Am	This work
pSEVAUO-M21402-CHI	φBT1	pUC	*SP43*-*CHI*-I5	-	Am	This work
pSEVAUO-M11701-NarBGC	φC31	pUC	NAR BGC	-	Ap-Am	This work
pSEVAUO-M21206F3′H-CPR	φBT1	pUC	*SF14*-*F3′H-CPR*	-	Ap-Tsr	OQ674225

## Data Availability

Not applicable.

## Supplementary Materials

The supporting information can be downloaded at: https://www.mdpi.com/article/10.3390/ijms24108879/s1.
