# Supplementary material for: Optimized De Novo Eriodictyol Biosynthesis in Streptomyces albidoflavus Using an Expansion of the Golden Standard Toolkit for Its Use in Actinomycetes
Source: Int J Mol Sci. 2023 May 17;24(10):8879. doi: 10.3390/ijms24108879 (PMC10219347; doi:10.3390/ijms24108879)
Supplement: Supplementary file 1 [file ijms-24-08879-s001.zip › ijms-2398713-supplementary.pdf]

Supplementary Figures

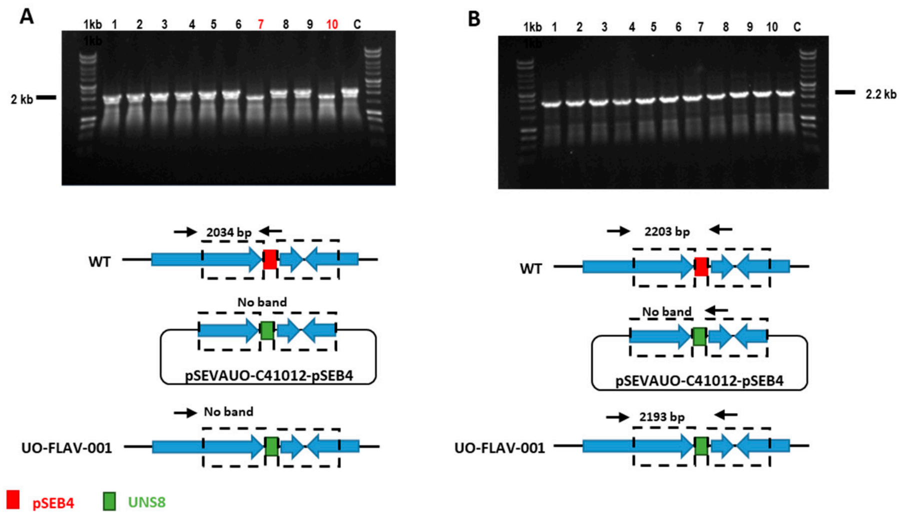

Figure S1: Generation of *S. albidoflavus* UO-FLAV-001 strain. A) Agarose gel and graphical representation of PCR verification of pseB4 replacement by UNS8 with primers pSEB4 del check fw and pSEB4 prot fw. Positive colonies are shown in red. B) Agarose gel and graphical representation of PCR amplification of a genome region containing the desired modification. Primers used were pSEB4 del check fw and pSEB4 del check rev. 1kb: PCRBIO Ladder II, C: *S. albidoflavus* J1074, WT: wild-type.

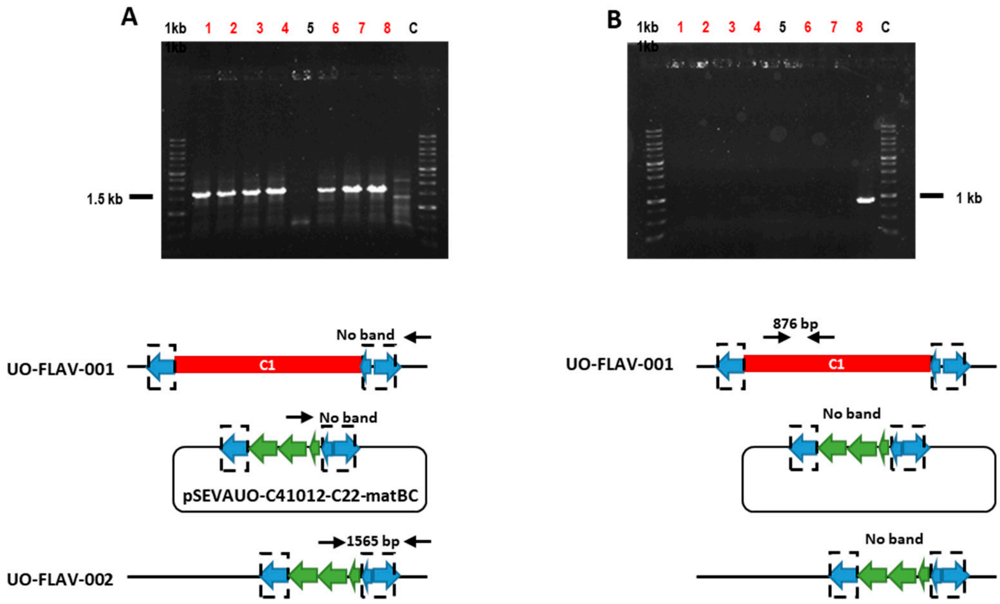

Figure S2: Generation of *S. albidoflavus* UO-FLAV-002 strain. A) Agarose gel and graphical representation of PCR verification of C1 replacement by P*ermE**-*matBC* with primers C1*matBC* check fw and C1*matBC* check rev. Positive colonies are shown in red. B) Agarose gel and graphical representation of PCR amplification of a genome region within C1 to check presence of non-edited DNA. Primers used were C1 check fw and C1 check rev. 1kb: PCRBIO Ladder II, C: *S. albidoflavus* UO-FLAV-001.

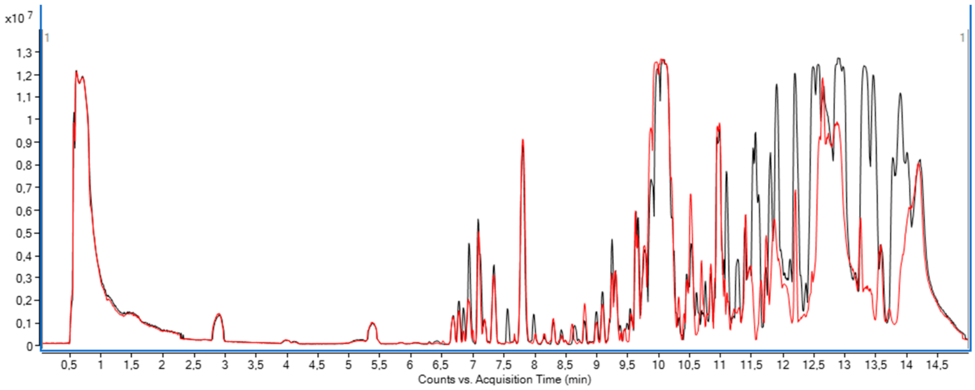

Figure S3: LC-HRESIMS base peak chromatograms (BPC) of *S. albidoflavus* WT (black) and *S. albidoflavus* UO-FLAV-002 (red) from cultures in R5A for 144 h. Antimycin peaks are shown.

Supplementary Tables

Table S1. Plasmids used in this work.

Plasmid	Use in this work	Reference or source
pCRISPomyces-2	Source of *pSG5* and *cas9-sgRNA*	[1]
pSG5c	Source of *pSG5c* with silent point mutations	This study
pCas9c	Source of *cas9c-sgRNA* with silent point mutations	This study
PCR™-Blunt II–TOPO®	Fragments sub-cloning	Invitrogen
pSETEc	Source of P_{ermE*} and *oriT-traJ*	[2]
pCR-Blunt-cas9c	Sub-cloning of *cas9c*	This study
pCR-Blunt-PermE*-cas9c	Source of P_{ermE*}-*cas9c-sgRNA*	This study
pSEVA88c1	Source of AmR, pUC and cargo	[3]
pGM1190	Source of TsrR nucleotide sequence	[4]
pSEVA181Thio	Source of TsrR	EXPLORA
pCRBluntTsr	Sub-cloning of TsrR	This study
pSEVA182	Source of ApR	[5]
pCRBluntAp-Tsr	Source of ApR-TsrR	This study
pSEVA23a1	Source of KmR	[3]
pSEVA181Hyg	Source of HygR	EXPLORA
pSEVA181BT1int	Source of φBT1 integrase	EXPLORA
pSEVAUO-21002	Receptor vector (*pUC-φBT1-Am*) for Cargo cloning	This study
pSEVA23g19g1	Source of cargo 1AI2	[6]
pSEVA23g19g2	Source of cargo 2AI3	[6]
pSEVA23g19g3	Source of cargo 3AI4	[6]
pSEVA23g19g4	Source of cargo 4AI5	[6]
pSEVA63g19gA	Source of cargo A13B	[6]
pSEVA63g19gB	Source of cargo B14C	[6]
pSEVA63g19gC	Source of cargo C15D and GmR	[6]
pSEVA28a1	Source of φC31 integrase	[3]
pPhiC31c	Source of φC31 integrase with silent point mutations	This study
pCRBluntAmR	Sub-cloning of AmR	This study

pCRBluntApR-AmR	Source of ApR-AmR	This study
pSEVAUO-21003	Receptor vector (*pUC-φBT1-Ap-Am*) for cargo cloning	This study
pCRBluntGm-Tsr	Source of GmR-TsrR	This study
pSEVAUO-11001	Receptor vector (*pUC-φC31-Ap-Am*) for cargo cloning	This study
pSEVA181pSAM2	Source of pSAM2 integrase	This study
pSEVA88c1-PermE*-Ind-HA	Source of HA for P_{ermE*} cloning upstream indigoidine BGC	This study
pmatBC	Source of *matBC*	EXPLORA
pSEVA28b-P_{ermE*}-APIGC	Source of *P_{ermE*}-RBS, TAL* and *4CL*	[3]
pSEVA181permE	Source of P_{ermE*} (Level 0 MoClo)	EXPLORA
pSEVA181SF14	Source of *SF14* (Level 0 MoClo)	EXPLORA
pSEVA181SP25	Source of *SP25* (Level 0 MoClo)	EXPLORA
pSEVA181SP43	Source of *SP43* (Level 0 MoClo)	EXPLORA
pSEVA181RiboJ-RBS	Source of RiboJ-RBS (Level 0 MoClo)	EXPLORA
pSEVA181TAL	Source of *TAL* cured (Level 0 MoClo)	This study
pSEVA1814CL	Source of *4CL* cured (Level 0 MoClo)	This study
pSEVA181CHS	Source of *CHS* (Level 0 MoClo)	EXPLORA
pSEVA181CHI	Source of *CHI* (Level 0 MoClo)	EXPLORA
pIDTSMARTttsbib	Source of *ttsbib* (Level 0 MoClo)	IDT
pSEVA181F3H-CPR	Source of F3’H-CPR chimaera	EXPLORA
pCRBluntAm-Tsr	Source of AmR-TsrR	This study
pSEVAUO-M21202F3H-CPR	Sub-cloning of F3’H-CPR transcription unit	This study

Table S2. Strains used in this work.

Strain	Use in this work	Reference or source
Escherichia coli Top10	Routine sub-cloning and DNA propagation	Invitrogen
Escherichia coli ET12567/pUZ8002	*oriT*-containing plasmids delivery to *S. albidoflavus* through conjugation	[7]
Streptomyces albidoflavus J1074	Source of DNA and parental strain for chassis generation and flavonoid production	[8]
S. albidoflavus M11701	*S. albidoflavus* J1074 with plasmid pSE-VAUO-M11701 integrated into φC31 site	This study
S. albidoflavus M21703	*S. albidoflavus* J1074 with plasmid pSE-VAUO-M21703 integrated into φBT1 site	This study
S. albidoflavus M31705	*S. albidoflavus* J1074 with plasmid pSE-VAUO-M31705 integrated into pSAM2 site	This study
S. albidoflavus UO-FLAV-001	*S. albidoflavus* J1074 with *pseB4* replaced by UNS8	This study
S. albidoflavus UO-FLAV-002	*S. albidoflavus* UO-FLAV-001 with antimcyin BGC-candicidin BGC-unknown NRPS/PKS BGC replaced by *PermE*-matBC*	This study
S. albidoflavus WT-NAR	*S. albidoflavus* J1074 with naringenin BGC integrated into φC31 site	This study
S. albidoflavus WT-ERI	*S. albidoflavus* WT-NAR with F3′H-CPR integrated into φBT1 site	This study
S. albidoflavus UO-FLAV-002-NAR	*S. albidoflavus* UO-FLAV-002 with naringenin BGC integrated into φC31 site	This study
S. albidoflavus UO-FLAV-002-ERI	*S. albidoflavus* UO-FLAV-002-NAR with F3′H-CPR integrated into φBT1 site	This study

Table S3. Primers used in this study.

Primer	Sequence 5′ – 3′
pSG5c F1 fw	ctgaaggtcctcaatcgcactggaaacatcaaggtcgctgTTTCCATAGGCTCCGCCC
pSG5c F1 rev	CGCCGGAAGGCCAACGAG
pSG5c F2 fw	ggccagctcctccgtgcgggcctcgttggccttccggcggGCCTCGCGGAAAGCAGCTTC
pSG5c F2 rev	tgcagctcgccgccgacggaCTCGCGGACGCCGGGTAC
pSG5c F3 fw	ccgtacccggcgtccgcgagtCCGTCGGCGGCGAGCTGC
pSG5c F3 rev	gaggcgttcaagggctgcggcACCCGGCTCACTGCCGGG
pSG5c F4 fw	tcccggcagtgagccgggtgCCGCAGCCCTTGAACGCC
pSG5c F4 rev	cgaccttgatgtttccagtgcgattgaggaccttcagtgcgctagcATGCCAGGATCAACAGGAC
cas9c F5 fw	tttgctgctccttcggtcgcACGTGCGTCTACGGGCAC
cas9c F2 rev	atgccctcggtcacgtacttcACCTTGGTCAGCTCGTTG
cas9c F3 fw	acaacgagctgaccaaggtgAAGTACGTGACCGAGGGC
cas9c F3 rev	aagccgccgtacttcttcggaTCCCAGTCCTTCTTCCGG
cas9c F4 fw bis	cccggaagaaggactgggaTccgaagaagtacggcggc
cas9c F4 rev	aggtgcccgtagacgcacgtgCGACCGAAGGAGCAGCAAAAAAAG
cas9c F2 fw	ggtggggtaaccaggctaacctcccgtaggaggacgacaATGGACAAGAAGTACAGCATC
cas9c F5 rev	ctctaacggacttgagtgaggttgtaaagggagttggctcTAAAAAACGCCCGGCGGC
CRISPR F1 UNS1 fw bis	CATTACTCGCATCCATTCTCAGGCTGTCTCGTCTCGTCTCCTTTTCCGCTGCATAACCC
CRISPR F1 UNS2 rev	GCTTGGATTCTGCGTTTGTTTCCGTCTACGAACTCCCAGCtcatggctctgccctcgg
CRISPR F2 UNS2 fw	GCTGGGAGTTCGTAGACGGAAACAAACGCAGAATCCAAGCGGCCGGCCctacgcgaccgctgtgtcga
CRISPR F3 UNS3 fw	GCACTGAAGGTCCTCAATCGCACTGGAAACATCAAGGTCGaggccaggaaccgtaaaa
CRISPR F3 UNS4 rev bis	GACTTTGCGTGTTGTCTTACTATTGCTGGCAGGAGGTCAGgggaccctctgaacaaatccagatg
CRISPR F4 UNS4 fw	CTGACCTCCTGCCAGCAATAGTAAGACAACACGCAAAGTCCGAGTGTCCGTTCGAGTGG
CRISPR F5Apra UNS5 fw	GAGCCAACTCCCTTTACAACCTCACTCAAGTCCGTTAGAGatttaaatCTCACGGTAACT
CRISPR F5Apra UNS1 rev	GAGACGAGACGAGACAGCCTGAGAATGGATGCGAGTAATGgaccgcCgtcTCAGCCAATCGACTG
UNS1 rev	GAGACGAGACGAGACAGCCTGAGAATG
UNS5 fw	GAGCCAACTCCCTTTACAACCTCACTC
cas9 D10A fw	gaagtacagcatcggcctggCcatcggcaccaacagcgtg
cas9 D10A rev	cacgctgttggtgccgatgGccaggccgatgctgtacttc
UNS2 fw	GCTGGGAGTTCGTAGACG
UNS3 rev	CGACCTTGATGTTTCCAG
Cargo UNS5 rev	ctctaacggacttgagtgaggttgtaaagggagttggctcgggaccctctgaacaaatccagatg
PhiC31 GA F2 fw	ccacccaggaaggcgtGttccgccagggcaacgtg
PhiC31 GA F2 rev	ccgttccgggtgatctccttCgtctcggacaccagctcg
PhiC31 GA F3 fw	cgagctggtgtccgagacGaaggagatcacccggaacgg
PhiC31 GA F3 rev	cccaggcggagctggcggtTttcttgccgatCgtctcgccgcgggtgg
PhiC31 GA F4 fw	ggcgagacGatcggcaagaaAaccgccagctccgcctggg
PhiC31 GA F4 rev	ctcccagagcagggccagCgtctcctcgtcgccctcg
PhiC31 GA F5 fw	cgagggcgacgaggagacGctggccctgctctgggag
PhiC31 GA F5 rev	cacgaacaggcccacgaaCacccgcttgtcgtccacg
PhiC31 GA F6 fw	cgtggacgacaagcgggtGttcgtgggcctgttcgtg

PhiC31 GA F1 rev	cacgttgccctggcggaaCacgccttcctgggtgg
PhiC31 GA F1 fw	CGACCTTGATGTTTCCAGTGCGATTGAGGACCTTCAGTGCGCTAGCcttcagacgtggcagatg
PhiC31 GA F6 rev	GCTGGGAGTTCGTAGACGGAAACAAACGCAGAATCCAAGCGGCCGGCCtcacgccgccacgtcctc
Ind Prot fw	ACGCatcgtgcgaggtgaactcat
Ind Prot rev	AAACatgagttcacctcgcacgat
Vector fw	AAGCTTGCGGCCGCGTCG
Vector rev	CCTAGGCGGCCTCCTGTG
Ind CRISPR F1 fw	gtcgccagggttttcccagtcacgacgcggccgcaagcttttcatggtgcccggcatc
Ind CRISPR F1 rev	ATCCTCCCCGCACCTCTCGCCAGCCGTCAAGATCGACTCCgcgtggtcggccctgttg
Ind CRISPR F2 fw	TGTGGGCACAATCGTGCCGGTTGGTAGGATCTAGCGTACTccatgagttcacctcgcac
Ind CRISPR F2 rev	attaaagcggataacaatttcacacaggaggccgcctaggaactccagcacctcgacg
pSEB4 prot fw	ACGCGAGAGCCGTACGTCTCGCCC
pSEB4 prot rev	AAACGGGCGAGACGTACGGCTCTC
pSEB4 F1 UNS7 fw	CAAGACGCTGGCTCTGACATTTCCGCTACTGAACTACTCGCCTCGCAGTACGACGTGG
pSEB4 F1 UNS8 rev	CCAGGTGGTTGATGGGTTGATTGCTTTGGTTGAGACGAGGGTCTCCGTCTACCCCCGC
pSEB4 F2 UNS8 fw	CCTCGTCTCAACCAAAGCAATCAACCCATCAACCACCTGGTTGTCGGTCCCGTCCCCT
pSEB4 F2 UNS6 rev	TATGTGACCGTAGAGTATTCTTAGGTGGCAGCGAACGAGTGTCGGTATCCATCGGCG
pSEVA-CRISPR UNS7 rev	CGAGTAGTTCAGTAGCGGAAATGTCAGAGCCAGCGTCTTGAGGCATCAAATAAAACGA
CRISPR F1 UNS1 fw bis	CATTACTCGCATCCATTCTCAGGCTGTCTCGTCTCGTCTCCTTTTCCGCTGCATAACCC
pSEVA-CRISPR UNS6 fw	CTCGTTCGCTGCCACCTAAGAATACTCTACGGTCACATACCTTGGACTCCTGTTGATA
C1 F2 fw	CACTCGAACGGACACTCGTGGGCGGTCTCCTTGAGGTC
C1 F2 rev	GTCGCCAGGGTTTTCCCAGTCACGACGCGGCCGCAAGCTTCAGACTCCGCCGGGAGAC
matBC rev	GGGACCTCAAGGAGACCGCCCACGAGTGTCCGTTCGAG
cas9c F1 rev	TGTCGTCCTCCTACGGGAG
matBC BglII GA fw	ccggttggtaggaggacgaagatctcgaggaggcagacaaatgtc
matBC fw	ACGCGCTGGTCCTTGCCGTCCATATGTTTTTTACACCAGGCC
C1 F1 fw	attaaagcggataacaatttcacacaggaggccgcctaggacctggatgtcgacggcg
C1 F1 rev	CCTGGTGTAAaaaaCATATGgacggcaaggaccagcgc
TAL GA F1 fw	GCGGCCGCGCGAATTCGAGCTCGGTACCCggtctcaAATGATGACCCTCCAGTCCCAGAC
TAL GA F1 rev	GTCCTGGGCGGGGTGGTCcTCGGGGCGGAGGTCGGC
TAL GA F2 fw	GCCGACCTCCGCCCCGAgGACCACCCCGCCCAGGAC
TAL GA F2 rev	GAAGCCGGACTGGAGGCCcGCCTGGCCACCGTGGAGG
TAL GA F3 fw	CCTCCACGGTGGCCAGGCgGGCCTCCAGTCCGGCTTC
TAL GA F3 rev	GCTGAGGGACTGGACGGAGACgGGGGTGGCGTTCGCGCG
TAL GA F4 fw	CGCGCGAACGCCACCCCcGTCTCCGTCCAGTCCCTCAGC
TAL GA F4 rev	TGCCTGCAGGTCGACTCTAGAGGATCCCCggtctcaAAGCctaggcgggggggtcgg
pSEVA181 fw	GGGGATCCTCTAGAGTCG
pSEVA181 rev	GGGTACCGAGCTCGAATT
4CL GA F1 fw	GCGGCCGCGCGAATTCGAGCTCGGTACCCggtctcaAATGatgttccggtcggagtacg
4CL GA F1 rev	gtcgcccttgcgcacgccCgtctccgccagtgcggc

4CL GA F2 fw	gccgcactggcggagacGggcgtgcgcaagggcgac
4CL GA F2 rev	TGCCTGCAGGTCGACTCTAGAGGATCCCCggtctcaAAGCtcagcggggctcgcggag
Prot seq rev	caaatccagatggagtaa
pSEB4 del check fw	AGCTGGACCAACGCCACC
pSEB4 del check rev	CGGCTCCACGTCGACCAC
C1matBC check fw	catctcccgcagctcctcc
C1matBC check rev	tgttgtcgatgcggatgaac
C1 check fw	atgcgcctggacctgaac
C1 check rev	TCACAGCCTGACATGGGTG
PermE fw	TCGATCTTGACGGCTGGC
PermE Ind check rev	caggaccccgagcatgtg
